# Supplementary material for: Probabilistic annotation of protein sequences based on functional classifications
Source: BMC Bioinformatics. 2005 Dec 14;6:302. doi: 10.1186/1471-2105-6-302 (PMC1361783; doi:10.1186/1471-2105-6-302)
Supplement: Additional File 1 — contains a more detailed description of the EC nomenclature to complement section "A database of enzymes" and the full calculation leading to equations(2) and(3) along with a figure illustrating their meaning. [file 1471-2105-6-302-S1.pdf]

Supplementary information for:

Probabilistic annotation of protein sequences based on functional classifications

Emmanuel D. Levy[1,*], Christos A. Ouzounis[1,§], Walter R. Gilks[2], Benjamin Audit[1,**,§]

[1]Computational Genomics Group, The European Bioinformatics Institute, EMBL Cambridge Outstation, Cambridge CB10 1SD, United Kingdom
[2]Medical Research Council Biostatistics Unit, Institute of Public Health, Cambridge CB2 2SR,United Kingdom

[*]Current address: Computational Genomics Group, MRC Laboratory of Molecular Biology, Hills Rd, Cambridge CB2 2QH, United Kingdom.
[**]Current address: Laboratoire Joliot-Curie and Laboratoire de Physique, CNRS UMR5672, Ecole Normale Supérieure, 46 Allée d'Italie, 69364 Lyon Cedex 07, France.

[§]Corresponding authors.

Email addresses:

EDL: elevy@mrc-lmb.cam.ac.uk
CAO: ouzounis@ebi.ac.uk
WRG: wally.gilks@mrc-bsu.cam.ac.uk
BA: audit@ebi.ac.uk

S1. The EC nomenclature

Enzymes are a group of a wide variety of proteins whose function is to accelerate the biochemical reactions i.e., they act as biological catalysts. They are precisely identified by the reaction they catalyse. The international Enzyme Commission1 (EC) has developed a functional classification scheme based on this observation. The scheme is hierarchical with four levels and can be represented as a tree. At the top level of the hierarchy, enzymatic activities are classified into six broad categories (oxidoreductases, transferases, hydrolases, lyases, isomerases or ligases). The three following levels provide finer details on the exact biochemical reaction catalyzed by an enzyme. In the current state, the EC nomenclature indexes 1819 enzymatic activities for which at least one protein catalysing the reaction is known. An EC number, composed of four fields separated by dots, uniquely identifies each EC class; each field is an integer value identifying a sub-branch of the preceding field. For instance, in the EC number 1.2.1.21, the first digit "1" means that the enzyme is an oxidoreductase, the second digit "2" corresponds to the sub-class of oxidoreductases acting on aldehyde or oxo- groups, the third digit "1" indicates that NAD or NADP is a cofactor of the reaction and the last digit "21" specifies that glycolaldehyde is the substrate of the enzyme. An EC number with only 1, 2 or 3 of the leftmost fields represent an internal node in the hierarchy and refers to all children EC classes of that node. For example, the EC number 1.- includes all oxidoreductases.

S2. Evaluating the probability of a protein sequence to belong to a functional class: an univariate Bayesian approach

Here, we propose to estimate, independently for each of the functional classes, the probability $P(\mathrm{c} \in \Omega_\mathrm{j} \mid \mathrm{Y}_{\Omega_j}^{(\alpha)}(c))$ for a protein c drawn at random, to belong to class Ω_j given $\mathrm{Y}_{\Omega_j}^{(\alpha)}(c)$ i.e. we estimate probabilities knowing one variable (indicator) only. Using Bayes theorem, we find:

$$P(\mathrm{c} \in \Omega_\mathrm{j} \mid \mathrm{Y}_{\Omega j}^{(\alpha)}(c)) = \frac{P(\mathrm{Y}_{\Omega_j}^{(\alpha)}(c) \mid \mathrm{c} \in \Omega_j) * P(\mathrm{c} \in \Omega_j)}{\sum_{k=1}^{n}\left[P(\mathrm{Y}_{\Omega_j}^{(\alpha)}(c) \mid c \in \Omega_k) * P(\mathrm{c} \in \Omega_k)\right]} = \frac{P(\mathrm{Y}_{\Omega_j}^{(\alpha)}(c) \mid c \in \Omega_j) * P(\mathrm{c} \in \Omega_j)}{P(\mathrm{Y}_{\Omega_j}^{(\alpha)}(c))}, \quad \text{(S1)}$$

where $P(\mathrm{c} \in \Omega_\mathrm{j})$ represents the relative size of class Ω_j and $P(\mathrm{Y}_{\Omega_j}^{(\alpha)}(c) \mid \mathrm{c} \in \Omega_j)$ is the probability density function of the correspondence indicators of proteins from Ω_j at the point $\mathrm{Y}_{\Omega_j}^{(\alpha)}(c)$. In other words, we propose to compare the correspondence indicator of a new protein c with class Ω_j to the set of equivalent indicators measured for the reference set of proteins that have a known functional class. This set of probabilities can be viewed as an alternative to the correspondence indicators, but additionally, their value is a direct measure of the confidence we can have in a functional assignment. In this work, we perform the functional annotation using the "highest probability" strategy. However, it is noteworthy that in a more realistic context, where proteins can belong to more than one functional class, these probabilities can be used to assign a new protein to zero, one or more functional classes.

---

[1] http://www.chem.qmul.ac.uk/iubmb/enzyme/

The estimation of the probability $P(c \in \Omega_j \mid Y^{(\alpha)}_{\Omega_j}(c))$ is performed as follows: $P(c \in \Omega_j)$ is simply estimated by:

$$P(c \in \Omega_j) = N_j / N, \tag{S2}$$

where $N = \sum_{k=1}^{n} N_k$ is the total size of the reference dataset. Also, $P(Y^{(\alpha)}_{\Omega_j}(c) \mid c \in \Omega_j)$ is estimated by the proportion of proteins that truly belong to Ω_j with a correspondence indicator comprised in an interval $[(Y^{(\alpha)}_{\Omega_j}(c) - \lambda), (Y^{(\alpha)}_{\Omega_j}(c) + \lambda)]$. In the same manner, $P(Y^{(\alpha)}_{\Omega_j}(c))$ is estimated by the proportion of proteins of the entire dataset hitting Ω_j with a correspondence indicator in that same interval. Thus,

$$\hat{P}(Y^{(\alpha)}_{\Omega_j}(c) \mid c \in \Omega_j) = N_{\Omega_j}(Y^{(\alpha)}_{\Omega j}(c) \pm \lambda) / N_j \tag{S3}$$

and
$$\hat{P}(Y^{(\alpha)}_{\Omega_j}(c)) = N(Y^{(\alpha)}_{\Omega_j}(c) \pm \lambda) / N, \tag{S4}$$

where $N_{\Omega_j}(Y^{(\alpha)}_{\Omega j}(c) \pm \lambda)$ and $N(Y^{(\alpha)}_{\Omega_j}(c) \pm \lambda)$ are respectively, the number of proteins truly belonging to class Ω_j and the number of proteins from the entire dataset, whose correspondence indicator with class Ω_j is comprised in $[(Y^{(\alpha)}_{\Omega_j}(c) - \lambda), (Y^{(\alpha)}_{\Omega_j}(c) + \lambda)]$.

We finally obtain an estimator of $P(c \in \Omega_j \mid Y^{(\alpha)}_{\Omega j}(c))$ by replacing the different terms in equation (S1) by their estimators using equations (S2), (S3) and (S4):

$$\hat{P}(c \in \Omega_j \mid Y^{(\alpha)}_{\Omega_j}(c)) = \frac{\frac{N_j(Y^{(\alpha)}_{\Omega j}(c) \pm \lambda)}{N_j} * \frac{N_j}{N}}{N(Y^{(\alpha)}_{\Omega j}(c) \pm \lambda) * \frac{1}{\mathrm{N}}} = \frac{N_{\Omega_j}(Y^{(\alpha)}_{\Omega j}(c) \pm \lambda)}{N(Y^{(\alpha)}_{\Omega j}(c) \pm \lambda)}. \tag{S5}$$

We find that $\hat{P}(c \in \Omega_j \mid Y^{(\alpha)}_{\Omega_j}(c))$ is simply the ratio between two numbers: i) the number of proteins truly belonging to Ω_j having their correspondence indicator comprised in $[(Y^{(\alpha)}_{\Omega_j}(c) - \lambda), (Y^{(\alpha)}_{\Omega_j}(c) + \lambda)]$ and ii) the number of proteins in the entire reference dataset having their correspondence indicator comprised in $[(Y^{(\alpha)}_{\Omega_j}(c) - \lambda), (Y^{(\alpha)}_{\Omega_j}(c) + \lambda)]$. If these two numbers are equal, it means that all the proteins from the dataset hitting Ω_j within $[(Y^{(\alpha)}_{\Omega_j}(c) - \lambda), (Y^{(\alpha)}_{\Omega_j}(c) + \lambda)]$ truly belong to Ω_j. In this case, the estimated probability for c to belong to Ω_j is 1. By contrast, if no protein from Ω_j hits Ω_j within the interval $[(Y^{(\alpha)}_{\Omega_j}(c) - \lambda), (Y^{(\alpha)}_{\Omega_j}(c) + \lambda)]$, the estimated probability for c belonging to Ω_j is 0. This mechanism is illustrated in Figure S1(a). λ is fixed for the annotation of each new protein such that the total number of sampled proteins $N(Y^{(\alpha)}_{\Omega j}(d) \pm \lambda)$ is always equal to 10. This can be viewed as an adaptive smoothing of the data: λ is increased until the interval $[(Y^{(\alpha)}_{\Omega_j}(c) - \lambda), (Y^{(\alpha)}_{\Omega_j}(c) + \lambda)]$ contains a predetermined quantity of information (10 proteins).
Note that an alternative way to estimate the probability density functions would have been to parametrise them. We found this approach impracticable for two major reasons: i) the shape

of density function changes from class to class (data not shown) and ii) the amount of data to fit the parameters is often very sparse: many classes have no more than 11 proteins.

S3. Determining the most likely functional class of a protein sequence: a multivariate Bayesian method of annotation.

Here, we propose to estimate the probability $P(c\in\Omega_j|\{Y_{\Omega_1}^{(\alpha)}(c)\ldots Y_{\Omega_n}^{(\alpha)}(c)\})$ of a new protein c to belong to Ω_j knowing the set $\{Y_{\Omega_1}^{(\alpha)}(c)\ldots Y_{\Omega_n}^{(\alpha)}(c)\}$ of its correspondence indicators with all the functional classes i.e. we estimate probabilities based on multiple variables (indicators). Using Bayes theorem, we find:

$$P(c\in\Omega_j|\{Y_{\Omega_1}^{(\alpha)}(c)\ldots Y_{\Omega_n}^{(\alpha)}(c)\}) = \frac{P(\{Y_{\Omega_1}^{(\alpha)}(c)\ldots Y_{\Omega_n}^{(\alpha)}(c)\}\,|\,c\in\Omega_j) * P(c\in\Omega_j)}{P(\{Y_{\Omega_1}^{(\alpha)}(c)\ldots Y_{\Omega_n}^{(\alpha)}(c)\})} \quad \text{(S6)}$$

where $P(\{Y_{\Omega_1}^{(\alpha)}(c)\ldots Y_{\Omega_n}^{(\alpha)}(c)\}\,|\,c\in\Omega_j)$ is the probability density function of the sets of correspondence indicators of proteins from class Ω_j at the point $\{Y_{\Omega_1}^{(\alpha)}(c)\ldots Y_{\Omega_n}^{(\alpha)}(c)\}$. In, the same manner as in the previous approach, we estimate $P(\{Y_{\Omega_1}^{(\alpha)}(c)\ldots Y_{\Omega_n}^{(\alpha)}(c)\}\,|\,c\in\Omega_j)$ and $P(\{Y_{\Omega_1}^{(\alpha)}(c)\ldots Y_{\Omega_n}^{(\alpha)}(c)\})$ by the proportion of proteins truly belonging to Ω_j or belonging to the entire dataset and having their set of correspondence indicators included in the sphere $B(\{Y_{\Omega_1}^{(\alpha)}(c)\ldots Y_{\Omega_n}^{(\alpha)}(c)\}, r)$ of radius r, centred at $\{Y_{\Omega_1}^{(\alpha)}(c)\ldots Y_{\Omega_n}^{(\alpha)}(c)\}$:

$$\hat{P}(\{Y_{\Omega_1}^{(\alpha)}(c)\ldots Y_{\Omega_n}^{(\alpha)}(c)\}\,|\,c\in\Omega_j) = N_j(\,B(\{Y_{\Omega_1}^{(\alpha)}(c)\ldots Y_{\Omega_n}^{(\alpha)}(c)\},\mathrm{r})\,)\,/\,N_j\,, \quad \text{(S7)}$$

and $$\hat{P}(\{Y_{\Omega_1}^{(\alpha)}(c)\ldots Y_{\Omega_n}^{(\alpha)}(c)\}) = N(\,B(\{Y_{\Omega_1}^{(\alpha)}(c)\ldots Y_{\Omega_n}^{(\alpha)}(c)\},\mathrm{r})\,)\,/\,N\,, \quad \text{(S8)}$$

where $N_j(\,B(\{Y_{\Omega_1}^{(\alpha)}(c)\ldots Y_{\Omega_n}^{(\alpha)}(c)\},\mathrm{r})\,)$ and $N(\,B(\{Y_{\Omega_1}^{(\alpha)}(c)\ldots Y_{\Omega_n}^{(\alpha)}(c)\},\mathrm{r})\,)$ are the numbers of proteins truly belonging to Ω_j or to the entire dataset, having a set of correspondence indicators included in the sphere $B(\{Y_{\Omega_1}^{(\alpha)}(c)\ldots Y_{\Omega_n}^{(\alpha)}(c)\}, r)$, respectively. We finally obtain an estimator of $P(c \in \Omega_j \mid \{Y_{\Omega_1}^{(\alpha)}(c)\ldots Y_{\Omega_n}^{(\alpha)}(c)\})$ by replacing the different terms in equation (S6) by their estimate using equation (S2), (S7) and (S8):

$$\hat{P}(c\in\Omega_j\,|\,\{Y_{\Omega_1}^{(\alpha)}(c)\ldots Y_{\Omega_n}^{(\alpha)}(c)\}) = \frac{N_j(\,B(\{Y_{\Omega_1}^{(\alpha)}(c)\ldots Y_{\Omega_n}^{(\alpha)}(c)\},\mathrm{r})\,)}{N(\,B(\{Y_{\Omega_1}^{(\alpha)}(c)\ldots Y_{\Omega_n}^{(\alpha)}(c)\},\mathrm{r})\,)}. \quad \text{(S9)}$$

In other words, the probability for c to belong to the class Ω_j is estimated as the ratio between the number of proteins within the sphere $B(\{Y_{\Omega_1}^{(\alpha)}(c)\ldots Y_{\Omega_n}^{(\alpha)}(c)\}, r)$ that truly belong to class Ω_j and the total number of proteins in the sphere. This mechanism is illustrated Figure S1(b).

As previously for λ, r is determined for each protein such that the total number of proteins sampled $N(\,B(\{Y_{\Omega_1}^{(\alpha)}(c)\ldots Y_{\Omega_n}^{(\alpha)}(c)\},\mathrm{r})\,)$ is always 10. Note that this method amounts to find the 10 closest proteins from the reference dataset to the point $\{Y_{\Omega_1}^{(\alpha)}(c)\ldots Y_{\Omega_n}^{(\alpha)}(c)\}$ in the correspondence indicator space. In order to reduce the computational cost of calculating the Euclidian distance to $\{Y_{\Omega_1}^{(\alpha)}(c)\ldots Y_{\Omega_n}^{(\alpha)}(c)\}$ for all the proteins in the reference dataset, we limit the search to those that hit at least one functional class also hit by the new protein.

	Number of Nodes			Number of proteins	
	**Lev. II**	**Lev. III**	**Lev. IV**	**A**	**B**
1 Oxidoreductases	21 (20)	75 (46)	449 (115)	7490	(5375)
2 Transferases	9 (9)	25 (22)	469 (184)	10432	(8456)
3 Hydrolases	9 (7)	45 (29)	563 (135)	8869	(6391)
4 Lyases	7 (6)	15 (13)	180 (75)	4432	(3477)
5 Isomerases	6 (6)	15 (12)	77 (27)	1827	(1475)
6 Ligases	5 (5)	9 (9)	81 (53)	3137	(2914)
Total	57 (53)	184 (131)	1819 (589)	35630	(28088)

Table S1: Summary statistics of the ENZYME database. For the 6 top-level enzyme categories of the EC hierarchy, we provide the number of protein members and the number of EC nodes of levels 2, 3 and 4 with at least one protein member. Values correspond to version 30 of the database. Numbers in parenthesis correspond to the statistics after filtering as described in Section A database of enzymes. Note that the total number of proteins (35630) is not equal to the sum over each top-level category because some proteins carry more than one EC number.

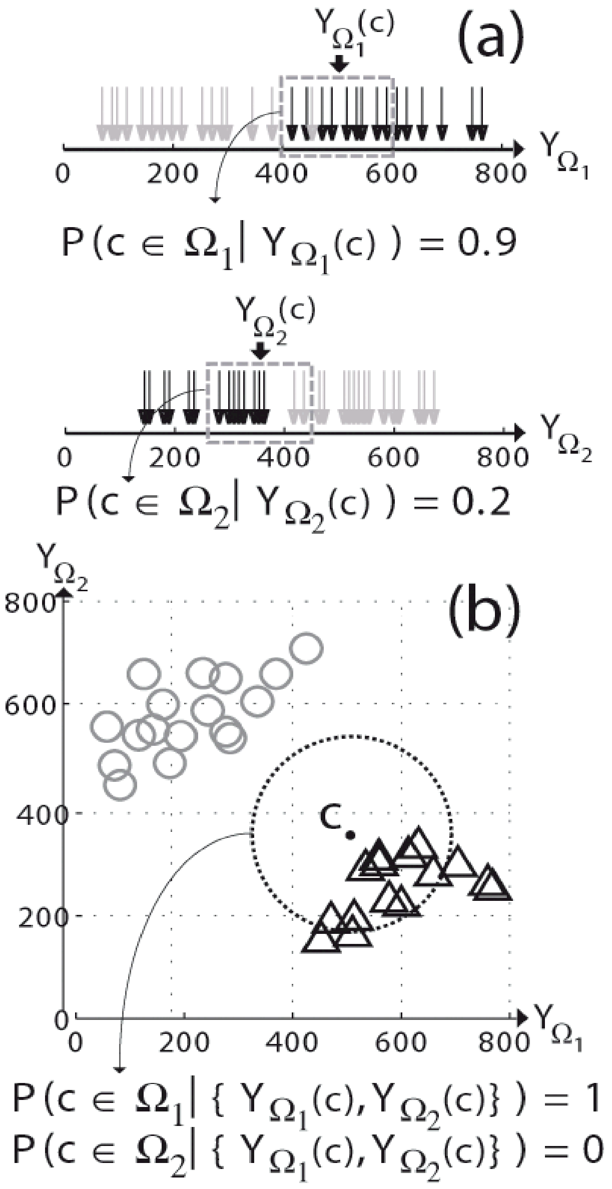

Figure S1: Illustration of the mechanism of calculation of the probabilities for a protein *c* to belong to functional classes Ω_1 and Ω_2 according (a) to the univariate Bayesian approach (Section S2; Eq. (S5)) and (b) to the multivariate Bayesian method (Section S3; Eq. (S9)). Probabilities are calculated according to the class membership of the 10 known proteins closest to *c*. In the univariate method, proximity is evaluated independently on each correspondence axis as illustrated by the two dashed rectangles in (a). In the case of the multivariate method, the sampling of characterised proteins is done only once globally as illustrated by the dashed circle in (b).
